# Stating clear indications for chest radiographs after cardiac surgery increases their efficacy and safely reduces costs

**DOI:** 10.1186/cc13441

**Published:** 2014-03-17

**Authors:** M Tolsma, TA Rijpstra, PM Rosseel, M Bentala, HA Dijkstra, NJ Van der Meer

**Affiliations:** 1 University Medical Center Utrecht, the Netherlands; 2 Amphia Hospital, Breda, the Netherlands

## Introduction

We investigated the efficacy and safety of chest radiographs (CXRs) performed on specified indications only, directly after cardiac surgery. CXRs in the ICU are frequently obtained routinely for postoperative cardiosurgical patients, despite the fact that the diagnostic and therapeutic efficacy of these CXRs is now known to be low [1]. Routine CXRs may only be beneficial for certain indications and the discussion regarding these indications and the safety of abandoning routine CXRs is still continuing [2].

## Methods

We prospectively included all patients who underwent major cardiac surgery in the year 2012. A direct postoperative CXR was performed only for certain specified indications. An on-demand CXR could be obtained during the postoperative period according to other specified indications. For all patients who did not have a CXR taken before the morning of the first postoperative day, a control CXR was then performed. All CXR findings were noted and classified, including whether or not they led to an intervention. Diagnostic and therapeutic efficacy values were calculated.

## Results

A total of 1,351 patients were included who mainly underwent coronary artery bypass grafting, valve surgery or a combination of both. Eighteen percent of patients underwent a minimally invasive cardiac surgery. The diagnostic efficacy for major abnormalities was clearly higher for the postoperative and on-demand CXRs, performed on indication, when compared with the next-morning routine CXR (6.7% and 6.9% vs. 2.9%) (*P *= 0.002). The therapeutic efficacy was also clearly higher for the postoperative and on-demand CXRs (2.9% and 4.1%), while the need for intervention after the morning control CXR was now reduced to be minimal (0.6%) (*P *= 0.002).

## Conclusion

Stating clear indications for CXRs following cardiac surgery increases the efficacy of these CXRs and safely reduces the total number of CXRs significantly.

## References

[B1] Tolsma M Kröner A van den Hombergh CL The clinical value of routine chest radiographs in the first 24 hours after cardiac surgery Anesth Analg 2011 112 139 142 10.1213/ANE.0b013e3181fdf6b7 21048091

[B2] Ganapathy A Adhikari NK Spiegelman J Scales DC Routine chest X-rays in intensive care units: a systematic review and meta-analysis Crit Care 2012 16 R68 10.1186/cc11321 22541022 PMC3681397

